# A tumour‐associated macrophage‐based signature for deciphering prognosis and immunotherapy response in prostate cancer

**DOI:** 10.1049/syb2.12097

**Published:** 2024-08-13

**Authors:** Jian Wang, Tao Guo, Yuanyuan Mi, Xiangyu Meng, Shuang Xu, Feng Dai, Chengwen Sun, Yi Huang, Jun Wang, Lijie Zhu, Jianquan Hou, Sheng Wu

**Affiliations:** ^1^ Department of Urology Affiliated Hospital of Jiangnan University Wuxi China; ^2^ Department of Urology The First Affiliated Hospital of Soochow University Suzhou China; ^3^ Department of Urology The First Affiliated Hospital of Nanjing Medical University Nanjing China

**Keywords:** cancer, diseases, patient diagnosis, patient treatment, pattern clustering, tumours

## Abstract

For the multistage progression of prostate cancer (PCa) and resistance to immunotherapy, tumour‐associated macrophage is an essential contributor. Although immunotherapy is an important and promising treatment modality for cancer, most patients with PCa are not responsive towards it. In addition to exploring new therapeutic targets, it is imperative to identify highly immunotherapy‐sensitive individuals. This research aimed to establish a signature risk model, which derived from the macrophage, to assess immunotherapeutic responses and predict prognosis. Data from the UCSC‐XENA, GEO and TISCH databases were extracted for analysis. Based on both single‐cell datasets and bulk transcriptome profiles, a macrophage‐related score (MRS) consisting of the 10‐gene panel was constructed using the gene set variation analysis. MRS was highly correlated with hypoxia, angiogenesis, and epithelial‐mesenchymal transition, suggesting its potential as a risk indicator. Moreover, poor immunotherapy responses and worse prognostic performance were observed in the high‐MRS group of various immunotherapy cohorts. Additionally, APOE, one of the constituent genes of the MRS, affected the polarisation of macrophages. In particular, the reduced level of M2 macrophage and tumour progression suppression were observed in PCa xenografts which implanted in Apolipoprotein E‐knockout mice. The constructed MRS has the potential as a robust prognostic prediction tool, and can aid in the treatment selection of PCa, especially immunotherapy options.

## Graphical abstract

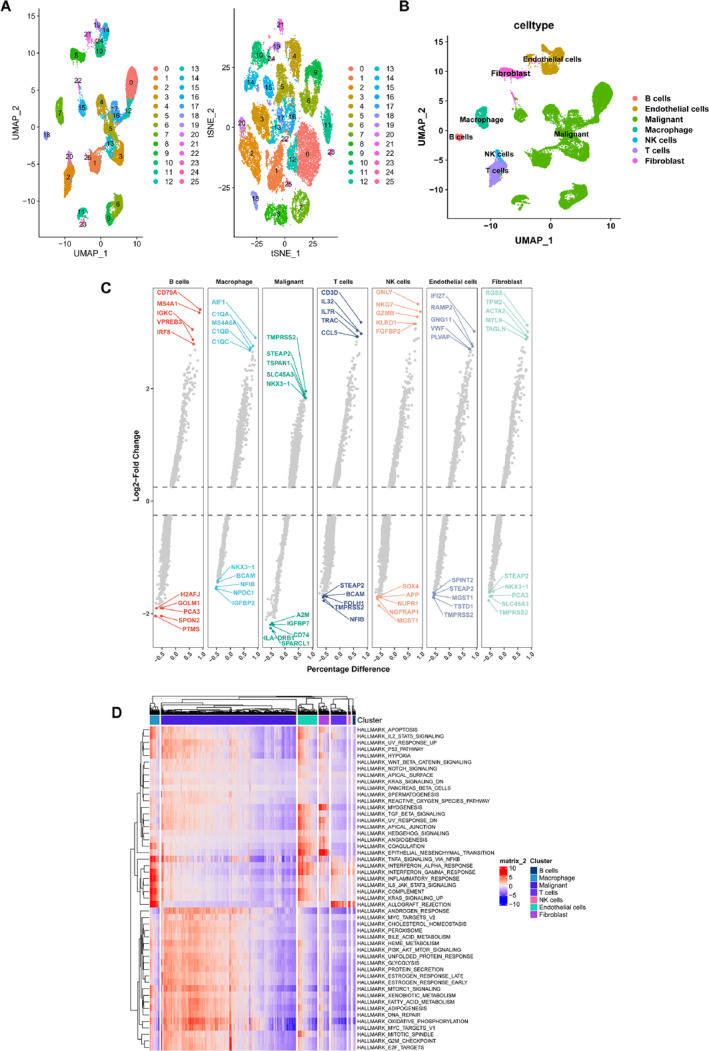

## 1 INTRODUCTION

As the second most diagnosed cancer among men worldwide [1], prostate cancer (PCa) is a common malignancy that seriously impacts male health. In China, the incidence and mortality rates of PCa have exhibited a remarkable upward trend in the past few years [2]. From 2014 to 2019, PCa incidence in the United States increased by 3% per year [3]. Although PCa patients have a high survival rate (97%) [3], their median survival time becomes extremely low when the castration‐sensitive prostate cancer (CSPC) progresses to castration‐resistant prostate cancer (CRPC) [4]. Conventional treatments for localised PCa include radical prostatectomy and radiation therapy, however, recurrence remains even after the initial treatment [5]. The mainstream treatment, androgen‐deprivation therapy (ADT), is only effective in patients with early‐stage PCa, and progression to CRPC is inevitable for most patients as sensitivity to ADT decreases [6]. Therefore, developing novel treatments, such as targeting circROBO1 and CENPA [7, 8], which might significantly prolong overall survival in PCa patients. In addition, immunotherapy are promising approaches for future clinical practice [9].

The immunotherapy of “cold” tumour PCa has been reported to be ineffective, owing to the heterogeneity of the tumour immune microenvironment (TIME). In the TIME, different immune cells play different roles in PCa progression [10]. The macrophage, an important mediator of tissue homoeostasis, has an essential regulatory role in immunotherapy response [11]. Moreover, the tumour‐associated macrophages (TAM) infiltration has been closely related to the poor prognosis of tumour patients [12]. Generally, the pro‐inflammatory M1 phenotype is featured with the strong tumoricidal activity [13], whereas the M2 macrophage possesses T cell‐suppressive capabilities, thereby shaping the suppressive TIME [14]. Furthermore, macrophages highly expressing iNOS can block the binding of T cell receptors and MHC through NO and its secondary production of peroxynitrite [15]. Meanwhile, immune checkpoints in TAM, including PD‐L1 and VTCN1, have been proven to inhibit T‐cell activation and proliferation [16, 17]. Certain molecules secreted by macrophages, such as IL‐10 and galectin‐3, could also exert direct inhibitory effects on CD8^+^ T cells [18, 19]. The indirect mechanisms of regulation include T cell recruitment and localisation in tumours [20]. Notably, animal experiments and clinical studies have indicated that TAM antagonists could enhance immunotherapy efficacy [11, 21].

As an important component of TIME, TAM plays a non‐negligible role in PCa progression and castration resistance [22]. There was a progressive increase in CD206‐positive macrophages from normal prostate to metastatic CRPC [23]. M2 TAM is enriched in PCa bone metastases and highly expresses CCL20, which induces the exhaustion of T lymphocytes through binding CCR6 [24]. HMGB1 induced by enzalutamide recruits and activates TAM. Subsequently, HMGB1‐activated TAM secretes IL6, thereby promoting treatment‐induced neuroendocrine differentiation [25]. Additionally, the level of TAM infiltration could serve as a powerful predictive tool for PSA failure and PCa progression [26].

In recent years, prediction models for PCa have been a hotspot [27, 28, 29]. Due to the profound impact of macrophage infiltration on PCa prognosis and immunotherapy response, this study aims to construct a robust risk score model based on macrophages. It also seeks to effectively distinguish individuals that are highly sensitive to immunotherapy. Accordingly, the constructed model could aid in the selection of immunotherapy strategies.

## 2 METHODS

### 2.1 Data source

Transcriptome sequencing cohorts, including The Cancer Genome Atlas (TCGA) pan‐cancer expression profiles, GSE54460, GSE61676, GSE78220, GSE116918, GSE91061, and GSE135222, were downloaded from the UCSC‐XENA (https://xenabrowser.net/datapages/) [30] and GEO database (https://www.ncbi.nlm.nih.gov/geo/) [31]. Meanwhile, we obtained single‐cell sequencing data (GSE120575, GSE123813, GSE141445, GSE145281, and PRIME‐CUT) from the TISCH database (http://tisch.comp‐genomics.org) [32], GEO database, and Mendeley Data (https://data.mendeley.com/). The IMvigor210 data was sourced from the *R* package “IMvigor210CoreBiologies” [33]. The Immune Cell Abundance Identifier (ImmuCellAI) database (http://bioinfo.life.hust.edu.cn/ImmuCellAI#!/) [34] provided data on tumour immune cell infiltration. Furthermore, the ComPPI database was employed for obtaining predicted protein–protein interaction (PPI) data [35].

Macrophages which are obtained through flow sorting from the mouse PCa tissues were used for library construction and RNA sequencing (HaploX Biotechnology, Shanghai). The platform used for library sequencing was Illumina PE150. After filtering out low quality sequences, we used the HISAT2 software to map the RNA‐seq reads to the mouse reference genome [36]. The obtained read counts were eventually converted to TPM.

### 2.2 Single‐cell data analysis

The Seurat tool (R package “Seurat”) was used for filtering and analysis of single‐cell data [37]. Cells that meet the following conditions were preserved: (1) Number of genes >200; (2) Number of genes <7500; (3) The proportion of mitochondrial gene expression <5%. We performed data normalisation, principal component analysis (PCA), uniform manifold approximation and projection (UMAP) and t‐distributed stochastic neighbour embedding (tSNE) dimensionality reduction, as per the manufacturer's instructions. The Seurat function “FindAllMarkers” was used to identify differentially expressed genes (DEGs) among different cell types. The *R* package “SingleR” [38] was an assistant tool for the cell annotation.

### 2.3 Gene set variation analysis and over‐representation analysis

To perform the gene set variation analysis (GSVA) [39], the Hallmark, Kyoto Encyclopaedia of Genes and Genomes (KEGG) [40] and Reactome datasets [41] were obtained from the molecular signatures database (MSigDB) [42], and each term was scored using the *R* package “GSVA”. In the meantime, we constructed the macrophage‐related score (MRS) through GSVA. The dataset used for the enrichment analysis included Gene Ontology (GO) and KEGG pathways (R package “ClusterProfiler”) [43].

### 2.4 Adjustment of batch effects

When different datasets were merged, the “limma” [44] and “sva” *R* packages were used for removing batch effects. Meanwhile, the PCA analysis to examine batch effects was performed using the *R* packages “FactoMineR” and “factoextra”.

### 2.5 Consensus clustering analysis

Unsupervised clustering is a method to perform the subgroup classification of the data. We implemented the cluster analysis and visualisation of the results with the *R* package “ConsensusClusterPlus” [45].

### 2.6 Analysis of the immune microenvironment, mutation, and chemotherapy response

To assess the heterogeneity and the composition of the tumour microenvironment, the *R* package “ESTIMATE” [46] was used. The Immune Score, Stromal Score, and ESTIMATE Score of each sample were estimated separately to elucidate the differences in the TIME. The CIBERSORT algorithm quantified the levels of the macrophage M1 and M2 cell infiltration within the tumour [47]. For gene mutation analysis and visualisation, the *R* package “maftools” [48] was then applied. The GSCA database helped visualise the summary of copy number variants (CNVs) [49]. We also used the *R* package “pRRophetic” [50] to predict the half maximal inhibitory concentration (IC50) values of each sample for various anti‐cancer drugs.

### 2.7 Animal model of prostate cancer

Male Apoe‐KO (Strain No. T0014588) and wide‐type (WT) C57BL/6 mice were obtained from GemPharmatech (Nanjing, China). The mice were kept in specific pathogen‐free animal houses (12‐/12‐h light/dark cycle; 45%–65% humidity; 20–24°C). C57BL/6 mice (6–8 weeks old) were injected with TRAMPC‐2 cells, which were infected with luciferase in situ (6 × 10^5^ cells each). Tumour sizes were monitored by detecting fluorescence. At sacrifice, mice were anesthetised by the inhalation of isoflurane and euthanised via cervical dislocation. Ultimately, tumour sizes were computed as follows: volume = 0.5 × Length × Width^2^.

### 2.8 Tissues dissociation and cell sorting

PCa tissues were isolated from the mice. Next, the tissues were minced and digested with the type IV collagenase (1 mg/ml), type II collagenase (2 mg/ml), and Y‐27632 (10 μM) (37°C; 1h). The dissociated tissue pellets were collected and further digested with TrypLE (Gibco, No. 12,605‐010). After digestion (37°C; 15 min), the cell suspensions were filtered through a 40 μm filter. Following this, the cells were sorted using flow cytometry with various antibodies (CD45‐APC, 1:200, BioLegend, No. 101,806; CD11b‐PE, 1:200, eBioscience, No. 12–0495‐82; F4/80, 1:200, BD Biosciences, No. 566,787; DAPI: 1 ng/mL).

### 2.9 Multiple immunofluorescence staining

Co‐staining of LYZ, TYROBP, and AIF1 respectively with CD68 was performed using a three‐colour Fluorescence kit (Recordbio Biological Technology, Shanghai, China) in human PCa tissues, which was based on the tyramide signal amplification (TSA) technology, according to the manufacturer's instructions. The primary antibodies were CD68 (1:200, Santa Cruz, No. sc‐20,060), LYZ (1:200, abclonal, No. A0641), TYROBP (1:200, Santa Cruz, No. sc‐166,084), and AIF1(1:200, abcam, No. ab178847). The stained sections were observed under a multichannel fluorescence imaging system, and images were acquired and analysed.

### 2.10 Statistical analysis

In this research, all analyses and visualisations were based on *R* (version 4.1.1). For continuous variables, the methods of comparison between the two groups included Student's *t*‐test and Wilcoxon rank‐sum test. When comparing categorical variables, the Chi‐squared test was the method used in the analysis. Simultaneously, the Kruskal–Wallis test was performed to compare the differences between the three groups. The correlation was calculated using Pearson's correlation analysis. Additionally, we also used the log‐rank test and Cox proportional hazard model to infer survival prognosis. Finally, *p* < 0.05 was used as the statistically significance threshold.

## 3 RESULTS

### 3.1 Immune microenvironment within PCa

**FIGURE 1:**
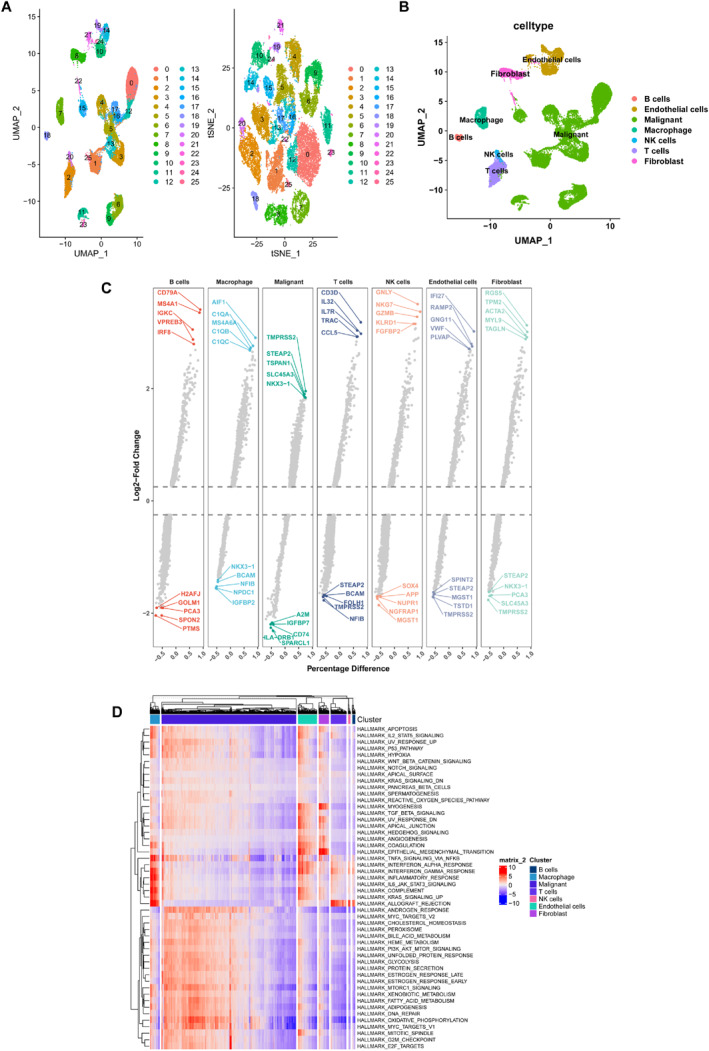
Overview of PCa tissue composition in GSE141445 (a) UMAP and tSNE clustering of cell populations (b) Identification of cell types (c) Highly and lowly expressed genes in each cell type and (d) Hallmark pathway differences among different cell types.

After processing the single‐cell data of PCa (GSE141445), a total of 26 clusters were classified (Figure 1a). We then divided all cells into B, endothelial, malignant, macrophage, NK, T, and fibroblast cells, according to the expression of markers in each cluster (Figure 1b). The DEGs of seven types of cells were shown in Figure 1c. It could be clearly observed that the macrophage was mainly enriched in immune‐related pathways, including TNFα signalling pathway via NF‐κB, interferon‐α response, interferon‐γ response, inflammatory response, IL‐6/JAK/STAT3 pathway, complement, and KARS signalling up (Figure 1d).

### 3.2 Identification of macrophage‐specific signature clusters

**FIGURE 2:**
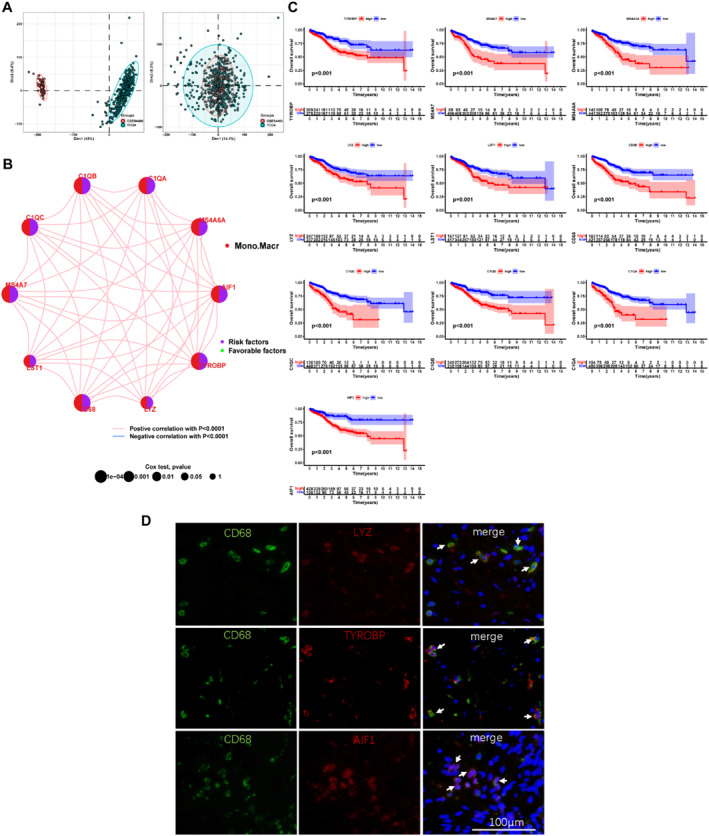
Comprehensive analysis of macrophage‐specific genes (a) PCA plots of TCGA‐PRAD and GSE54460 before and after batch effect removal (b) Correlations among macrophage‐specific gene expressions and (c) Prognostic analysis of 10 signature genes (d) LYZ, TYROBP, and AIF1 were co‐localised with CD68 in human PCa tissues.

We removed the batch effect when integrating TCGA‐PRAD and GSE54460 into a merged cohort (Figure 2a). Subsequently, the top 10 macrophage‐specific expressed genes were used as the signature genes, including TYROBP, MS4A7, MS4A6A, LYZ, LST1, CD68, C1QC, C1QB, C1QA, and AIF1. Notably, positive co‐expression networks were observed among these 10 genes (Figure 2b). Moreover, all macrophage‐specific genes were considered risk genes according to log‐rank tests (Figure 2c). In order to further verify the specific expression of the genes concentrated in macrophages, we verified the colocalisation of LYZ, TYROBP, AIF1, with CD68 respectively using the multicolour immunofluorescence method (Figure 2d). We employed the TISCH database to observe the expression of these genes in various single‐cell data of PRAD and found that they were also highly expressed in macrophages (Supplementary Figure 1). Furthermore, the GSVA scores of the 10 genes had a significantly positive correlation with macrophages in all 32 TCGA cancer cohorts (Supplementary Figure 2).

**FIGURE 3:**
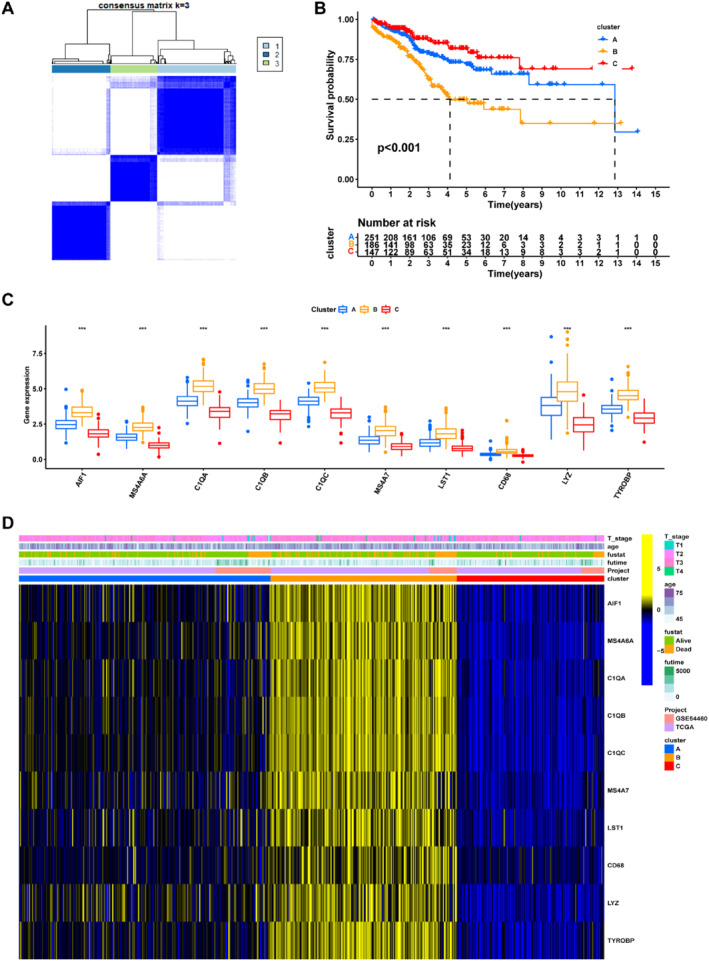
Cluster identification according to the macrophage‐specific signatures (a) Consensus matrix with *k* = 3 (b) Kaplan–Meier analysis showing prognostic differences among macrophage‐specific signature clusters (c) Boxplot demonstrating differences of signature genes and (d) Differences in clinicopathological features and macrophage‐specific genes. *p < 0.05; **p < 0.01; ***p < 0.001.

The unsupervised clustering method was used to classify the merged cohort into the most suitable three macrophage‐specific signature clusters (MSSC) based on the 10 signature genes (Figure 3a). The Kaplan–Meier plot demonstrated that the MSSC C had the best prognosis, while the MSSC B displayed the worst prognosis (Figure 3b), which was consistent with the signature gene expressions among different clusters. Furthermore, all signature genes were highly expressed in the MSSC B, compared to the other clusters (Figure 3c–d).

**FIGURE 4:**
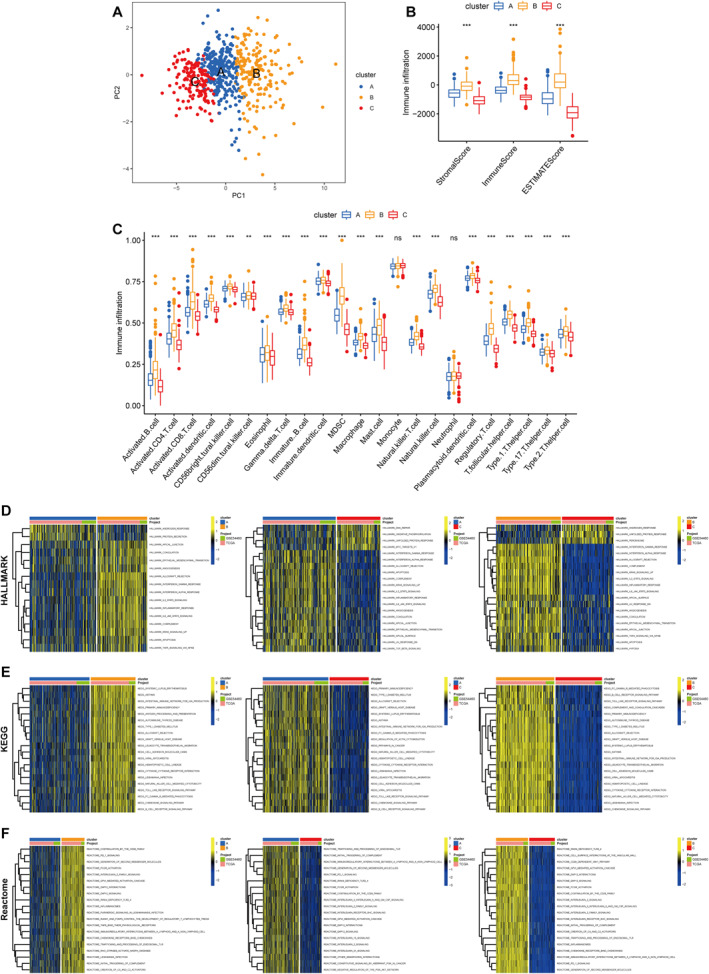
Cluster identification according to the macrophage‐specific signatures (a) PCA diagram of the merged cohort (b) Differences in Immune Score, Stromal Score, and ESTIMATE Score (c) Heterogeneity of the immune microenvironment among the three clusters and (d)‐(f) Comparisons of immune‐related pathways through Hallmark, KEGG, and Reactome datasets. *p < 0.05; **p < 0.01; ***p < 0.001.

In the PCA plot, the three MSSC clusters were well differentiated (Figure 4a). It was apparent that MSSC B with good prognosis had the highest Immune Score, Stromal Score and ESTIMATE Score (Figure 4b). In the meantime, the abundances of diverse immune cells (activated B cell, activated CD4^+^ T cell, activated CD8^+^ T cell, activated dendritic cell, macrophage, natural killer T cell, and natural killer cell) were also significantly increased in the MSSC B (Figure 4c). There were numerous pathway differences among the three clusters based on the Hallmark, KEGG, and Reactome databases (Figure 4d–f). In particular, MSSC B was enriched in epithelial‐mesenchymal transition (EMT), angiogenesis, hypoxia, primary immunodeficiency, and PD‐1 signaling pathways.

### 3.3 Classification of macrophage‐related clusters (MRCs) and construction of the MRS

**FIGURE 5:**
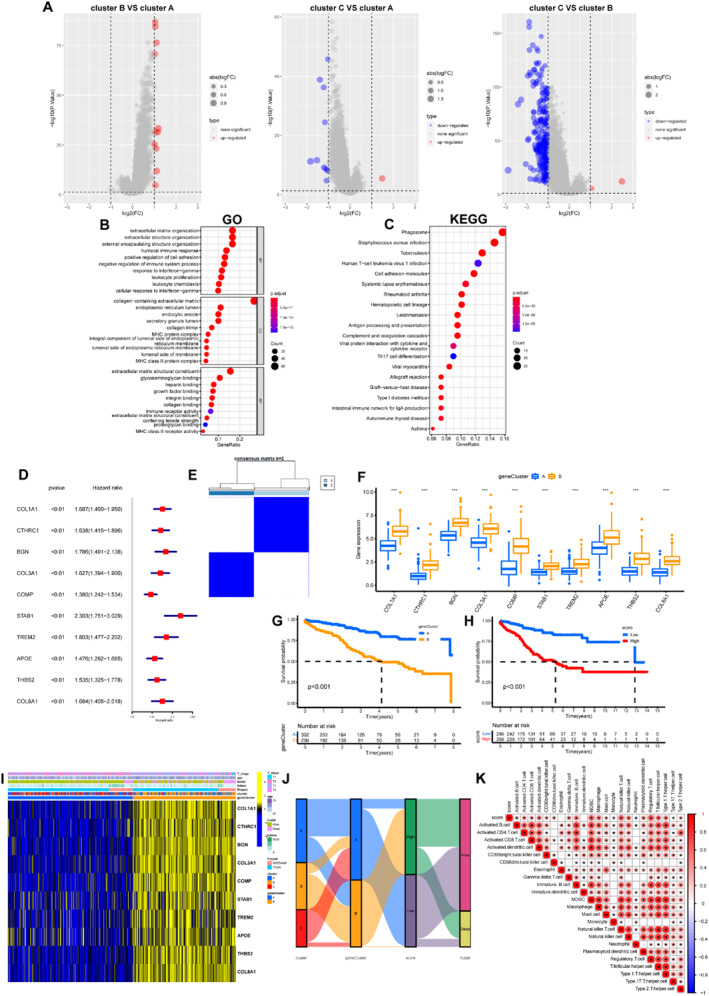
Establishment of MRCs and MRS (a) Analysis of differential gene expression (b)‐(c) GO and KEGG enrichment analysis of DEGs (d) The hazard ratios for the top 10 DEGs (e) Consensus matrix with *k* = 2 (f) Comparison of risk genes between different macrophage‐related clusters (g) Kaplan–Meier analysis showing prognostic differences between the MRC A and MRC B (h) Kaplan–Meier analysis showing prognostic differences between the high‐MRS and low‐MRS groups (i) Differences in clinicopathological features and selected genes and (j) Distribution of MSSCs, MRCs, MRS, and BCR outcomes (k) Correlations between MRS and immune cell infiltrations. *p < 0.05; **p < 0.01; ***p < 0.001.

We performed differential gene expression analysis between the three clusters, and subsequently obtained 299 DEGs, according to the set thresholds (|logFC| > 1, *p*‐value <0.05) (Figure 5a). These DEGs were mainly enriched in the extracellular matrix tissue, collagen‐containing extracellular matrix, extracellular matrix structural constituent, and phagosome (Figure 5b–c). The acquired DEGs were performed univariate Cox regression analysis, and the top 10 genes were selected for subsequent analysis based on *p*‐values.

Astonishingly, the selected 10 genes were all risk genes for PCa prognosis (COL1A1, CTHRC1, BGN, COL3A1, COMP, STAB1, TREM2, APOE, THBS2, and COL8A1) (Figure 5d). Consensus clustering based on the selected genes was performed, with the two macrophage‐related clusters being the most appropriate (Figure 5e–g). At the same time, we constructed the MRS with selected genes, and it was shown that elevated score portended a poor prognosis (Figure 5h). In addition, MRS also functioned as a prognostic predictor in the external cohort GSE116918 (Supplementary Figure 3). Obviously, the MRC B highly expressed all selected genes, and possessed a higher MRS (Figure 5i–j). Figure 5k demonstrated significant positive associations of MRS with myeloid‐derived suppressor cells (MDSC), macrophages and regulatory T cells (Treg). The frequency of mutations was significantly higher in patients with high MRS than those with low MRS (Supplementary Figure 4a–b), and the differentially mutated genes included RYR2, DCAF4L2, TP53, ADAMTS19, CDH12, DNAH11, DOCK10, FAM47A, FLG2, NELL1, OR4D5, and SETD2 (Supplementary Figure 4c).

### 3.4 Associations of the MRS with immune microenvironment

**FIGURE 6:**
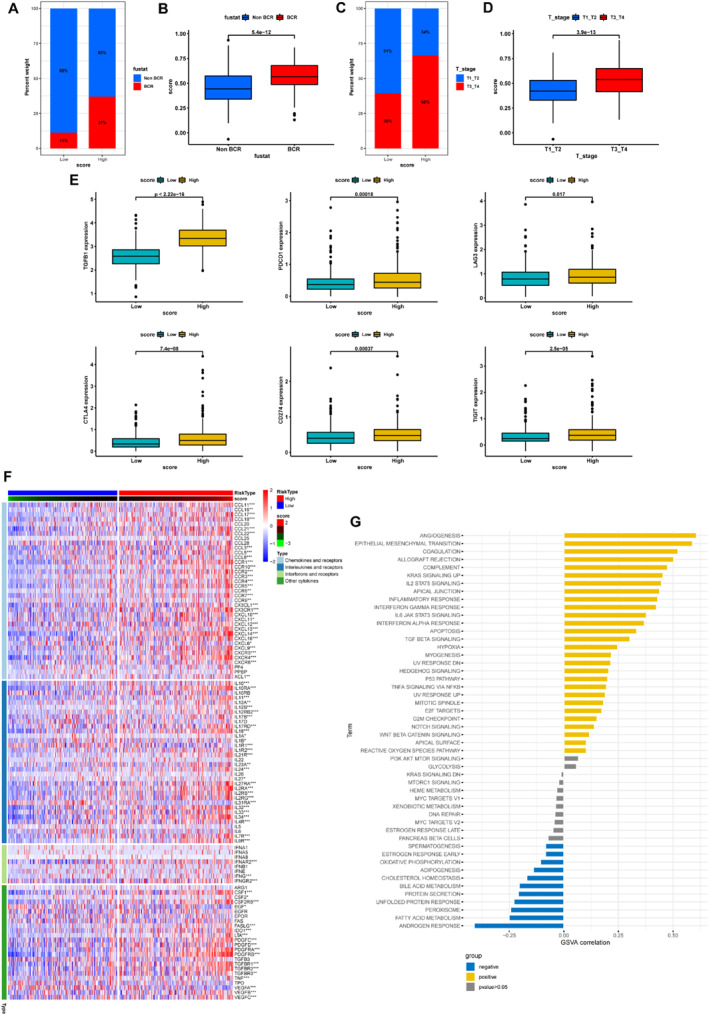
Comparisons of the immune microenvironment (a‐b) Association between MRS and BCR outcomes (c‐d) Association between MRS and T stages (e) Differences in immune checkpoints between different MRS groups (f) Differences in immune‐related genes between different MRS groups and (g) Correlations of MRS with immune‐related pathways. *p < 0.05; **p < 0.01; ***p < 0.001.

Both the biochemical recurrence (BCR) group and the advanced patients with T3‐4 possessed the elevated MRS (Figure 6a–d). Not only were there tumour cell differences between the high‐MRS and low‐MRS groups, but also significant alterations in the immune microenvironment. However, further researches will be necessary to explore the correlation between MRS and TIME. Firstly, the expression of immunosuppressive genes, including TGFβ1, PDCD1, LAG3, CTLA4, CD274, and TIGIT, was significantly higher in the high‐MRS group than in the low‐MRS group (Figure 6e). Secondly, the high‐MRS group was accompanied by the high expression levels of most chemokines, interleukins, interferons, other cytokines and receptors (Figure 6f). Correlation analysis showed a positive correlation with MRS for enriched terms, such as angiogenesis, EMT, hypoxia, and others (Figure 6g).

### 3.5 Application of MRS in the prediction of immunotherapy and chemotherapy response

**FIGURE 7:**
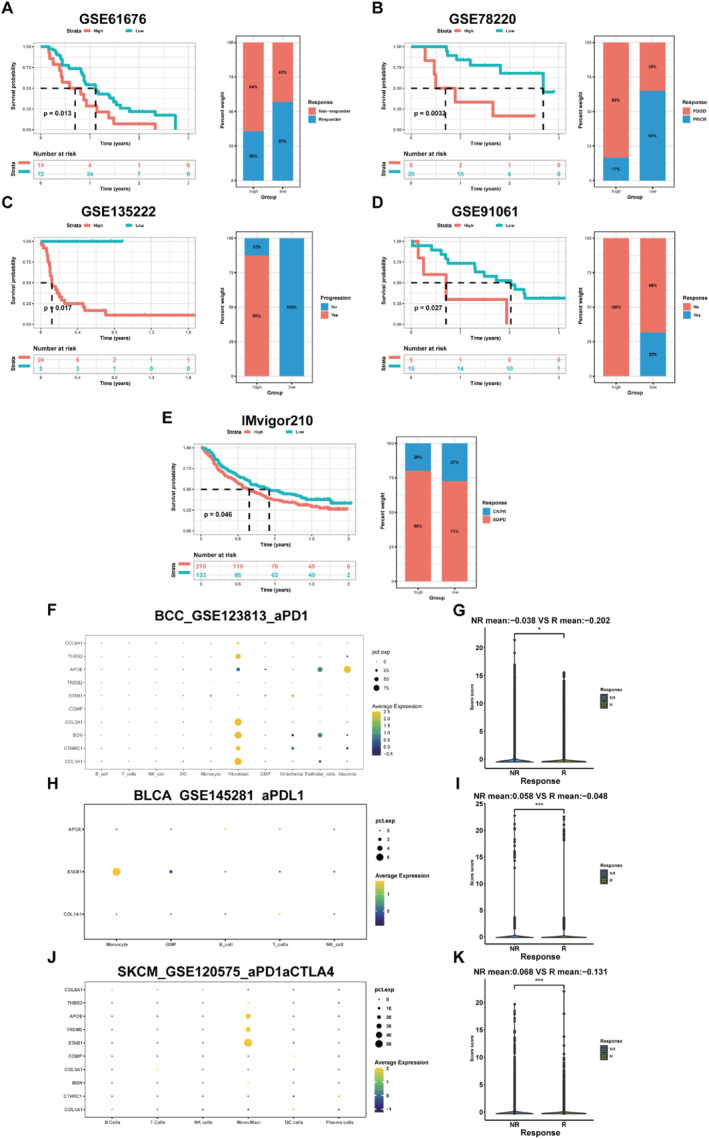
Application of the MRS to predict response to immunotherapy (a‐e) Prognostic differences and variations in immunotherapy response between the high‐MRS and low‐MRS groups in GSE61676, GSE78220, GSE135222, GSE91061, and IMvigor210 cohorts (f‐k) Distribution of selected genes and MRS differences between different immunotherapy responses in GSE123813, GSE145281, and GSE120575. *p < 0.05; **p < 0.01; ***p < 0.001.

In various immunotherapy cohorts, the prognosis was commonly poor in the high‐MRS group (GSE61676: non‐small cell lung cancer, combined targeted therapy of bevacizumab plus erlotinib; GSE78220: melanoma, anti‐PD‐1; GSE135222: non‐small cell lung carcinoma, anti‐PD‐1/PD‐L1; GSE91061: melanoma, nivolumab; IMvigor210: urothelial cancer, anti‐PD‐1) (Figure 7a–e). Moreover, immunotherapy was more effective in the low‐MRS group than that in the high‐MRS group. Also, MRS was calculated in the single‐cell data using GSVA, and we compared the differences of MRS among patients with different immunotherapy responses in the three types of tumours (GSE123813: basal cell carcinoma, anti‐PD‐1; GSE145281: bladder cancer, anti‐PD‐L1; GSE120575: melanoma, anti‐PD‐1/CTLA4) (Figure 7f–k). The genes that composed the MRS were primarily expressed in macrophages, monocytes, and fibroblasts. Meanwhile, it was apparent that immunotherapy non‐responders had a high MRS in all three tumours, consistent with the previous analysis of the immunotherapy cohort. Additionally, T cells exhibited lower levels, while the proportion of monocytes and macrophages was commonly higher in the high‐MRS group (Supplementary Figure 5). Thus, these findings indicated that the MRS could be a well‐predictive application for immunotherapy response in different cancers. As patients with high MRS were not sensitive to immunotherapy, it is imperative to find their suitable chemotherapeutic agents. According to speculation, drugs such as AICAR and ATRA were more effective in the high‐MRS group (Supplementary Figure 6).

**FIGURE 8:**
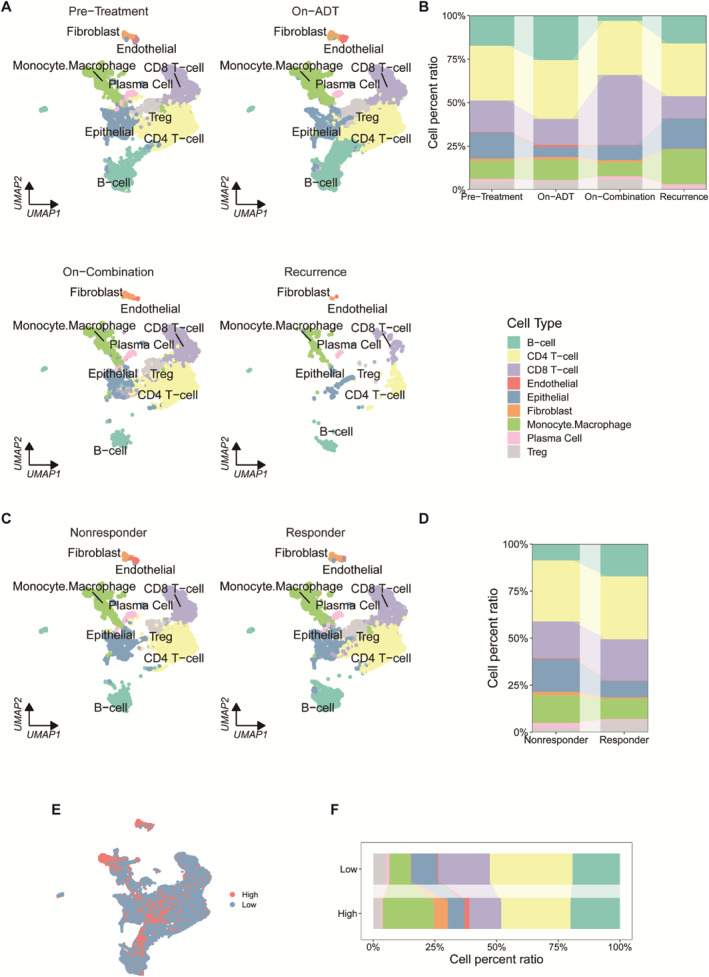
Application of the MRS in predicting PCa immunotherapy response (a) UMAP plot of scRNA‐seq data, including pre‐treatment, on‐ADT, on‐Combination, and recurrence (b) Relative frequencies of different cell lineages, separated by treatment time points (c) UMAP plot of scRNA‐seq data, including non‐responder and responder (d) Relative frequencies of different cell lineages, separated by treatment responses (e) Distribution of high‐MRS group and low‐MRS group in scRNA‐seq data and (f) Relative frequencies of different cell lineages, separated by MRS.

In an immunotherapy cohort which collected pre‐ and post‐treatment PCa samples [51], the combination therapy of ADT and anti‐PD‐1 resulted in an increase in the proportion of CD8^+^ T cells and a decrease in the proportion of monocytes and macrophages (Figure 8a–b). Relative to non‐responders, responders were found to have a higher percentage of B cells and CD8^+^ T cells, and a lower percentage of monocytes and macrophages (Figure 8c–d). There was a higher proportion of monocytes and macrophages and a lower proportion of CD4^+^ and CD8^+^ T cells in the high‐MRS group, compared to the low‐MRS group (Figure 8e–f).

### 3.6 Impact of APOE on PCa progression and M2 polarisation

**FIGURE 9:**
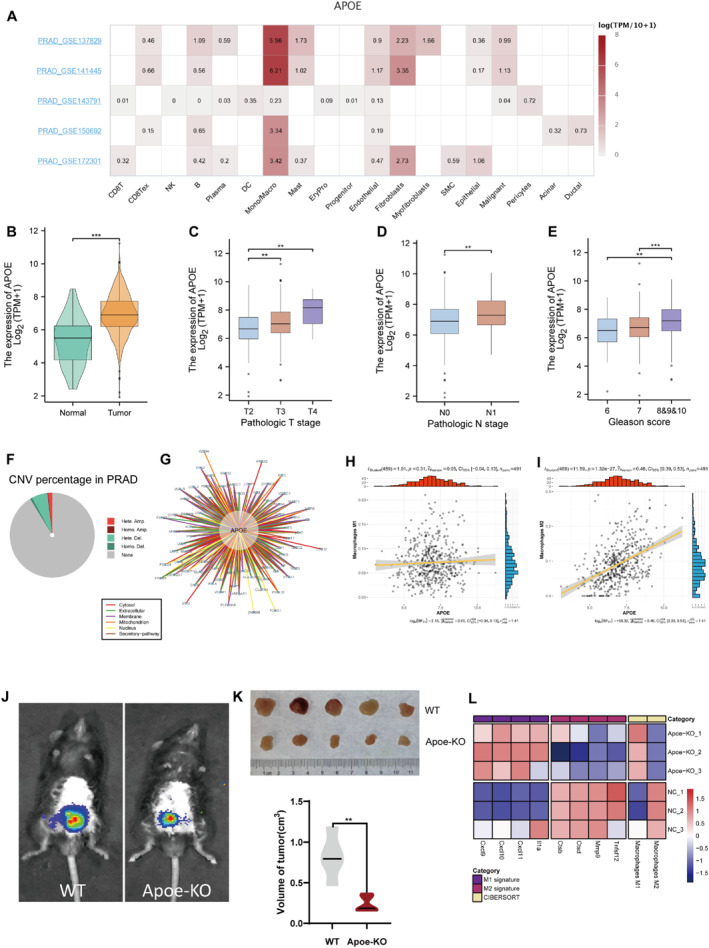
Summary of the function of APOE in PCa (a) Distribution of APOE in different cells (b) Comparison of APOE expression in PCa and normal prostate (c)‐(e) Variations of APOE expression in different pathological T stages, pathological N stages, and Gleason scores (f) The proportion of different CNVs in prostate cancer (g) APOE‐centred protein–protein interaction network (h)‐(i) Correlation analysis of APOE expression and macrophage M1 and M2 (j) TRAMPC‐2 cells, which were infected with luciferase, were injected into the prostate of C57BL/6 mice (WT or Apoe‐KO) in situ and representative bioluminescent images were taken on week 3 (k) The comparison of tumour volumes on week 3 and (l) Macrophage M1 and M2 differences in Apoe‐KO and wild‐type mouse. The values of M1 and M2 macrophage genes were represented with log_2_ (TPM+1), and the infiltration levels of M1 and M2 were calculated by the CIBERSORT algorithm. The final values were scaled. *p < 0.05; **p < 0.01; ***p < 0.001.

Among the genes that contribute to the MRS, we could apparently observe high expression of APOE in macrophages and monocytes of PCa (Figure 9a). APOE plays a carcinogenic role in both the tumourigenesis and progression of PCa (Figure 9b–e). Copy number profiling demonstrated that CNVs of APOE were mainly manifested as the heterozygous deletion (Figure 9f). The interaction network of APOE with proteins in different subcellular localisation also indicated its potential interaction linkages (Figure 9g). There is a clearly significant positive correlation between APOE and M2 macrophages in the TCGA‐PRAD cohort (Figure 9h–i).

To further explore the function of APOE, we established in vivo tumour models by injecting TRAMPC‐2 PCa cell lines into C57BL/6 mice (WT or Apoe‐KO), and found that the tumours in the Apoe‐KO group were relatively smaller (Figure 9j–k). We further sorted out macrophages for RNA‐seq and found that macrophages in the Apoe‐KO group were characterised with reduced M2 macrophage levels, compared with the control group (Figure 9l).

## 4 DISCUSSION

There have not been many successful clinical trials of immunotherapy for PCa [52, 53], and the only immunotherapy with a survival benefit for metastatic CRPC is Sipuleucel‐T therapy [54]. The immunosuppressive component is the primary reason why the “cold” tumour PCa is generally insensitive to immunotherapy [55]. As the most abundant cell population in various tumours, macrophages have the suppressive effect on the immune microenvironment, in addition to directly promoting AR signalling in PCa cells [56]. However, there exist certain macrophages associated with the improved prognosis, such as the zinc transporter‐expressing macrophage cluster [57]. Existing clinical indicators for risk assessment and treatment selection for PCa patients leave much to be desired [58]. Consequently, the MRS aimed to more accurately predict prognosis and response to immunotherapy were constructed.

In the previous report, a TAM‐associated gene signature demonstrated accurate predictive power in immunotherapy response for triple‐negative breast cancer [59]. For other types of breast cancer, the macrophage marker signature provided a reliable reference for immunotherapy selections [60]. Moreover, an M2 MRS comprising four hub prognostic genes was effective in predicting colon cancer prognosis, with high scores indicating more suitability for immunotherapy [61]. To the best of our knowledge, this study established an MRS with the predictive capacity of prognosis and immunotherapy in PCa for the first time. Given the co‐expression of M1 and M2 signals in the whole macrophages and the difficulty in distinguishing between M1 and M2 types [62], we did not further classify macrophages. We constructed the MRS and select multiple tumour data to validate the predictive ability of MRS for immunotherapy response. Patients with higher MRS possessed a higher proportion of the monocytic lineage, and a lower proportion of T cells in the single‐cell data, which is consistent with the acknowledged notion that macrophages are capable of directly or indirectly suppressing T cells [11]. The findings showed an overall better prognosis and more effective immunotherapy response in the low‐MRS group of non‐small cell lung cancer, melanoma, and urothelial cancer than that in the high‐MRS group, indicating the pan‐cancer applicability of the MRS capability. We are convinced that the reliable MRS would perform well in the prediction of PCa immunotherapy response in clinical settings.

Strikingly, there were significant positive correlations between the hypoxia, angiogenesis and MRS. Hypoxia, a common feature of solid tumours, plays an essential role in immune plasticity and hinders immunotherapy efficacy [63, 64]. The high expression of HIF1 in hypoxic regions, can recruit immature myeloid cells which are subsequently partially transformed into TAMs and also promote TAM retention [65, 66]. Moreover, M2 macrophages are more enriched in the intratumoral hypoxic environment [67]. This phenomenon is highly pro‐angiogenic, which is per the positive relationship results. In addition, EMT is the second most positively correlated Hallmark enriched term with MRS. Intriguingly, Huang et al. reported that the TAM‐derived CCL5 has a promoting effect on EMT for PCa cells [68].

APOE, a gene selected in the construction of MRS, is a macrophage‐specific gene and highly expressed in PCa. Previous reports have confirmed the tumourigenic and metastatic capabilities of APOE in pancreatic, breast, and lung cancers [69, 70, 71]. Zheng et al. found that Apoe was highly expressed in M2 macrophage‐derived exosomes. These exosomes reshape the cytoskeleton of gastric cancer cells and thereby favour cancer metastasis [72]. Additionally, a reduction in the M2 phenotype was observed in gastric carcinoma, colorectal carcinoma, and hepatocellular carcinoma of Apoe knockout mice [73]. In our research, the loss of Apoe resulted in the decreased abundance of M2 macrophages within PCa tissues. Previous experiments in mice have indicated that APOE inhibitors have an enhancing effect on the efficacy of immunotherapy [73]. Hence, we hypothesise that the combination of APOE inhibitors and immunotherapy could be a feasible clinical strategy.

Certainly, the absence of real‐world validation of the predictive efficacy for prognosis and immunotherapy response of the MRS is a limitation of this study. Beyond that, the specific mechanism of how APOE affects macrophage polarisation requires elucidation. Overall, MRS was identified as a robust indicator in predicting BCR and immunotherapy response. It is reasonable to speculate that MRS will hold a great promise in the clinical application of PCa.

## AUTHOR CONTRIBUTIONS

Jian Wang, Tao Guo, and Yuanyuan Mi contributed equally to this work. Conceptualisation: Jian Wang, Tao Guo, and Yuanyuan Mi. Investigation: Jian Wang, Tao Guo, Yuanyuan Mi, and Xiangyu Meng. Methodology, software, visualisation, formal analysis, and data curation: Xiangyu Meng, Shuang Xu, Feng Dai, Chengwen Sun, Yi Huang, Jun Wang, Lijie Zhu, Jianquan Hou, and Sheng Wu. Writing‐original draft: Jian Wang, Tao Guo, Jianquan Hou, and Sheng Wu. Writing‐review & editing: Jian Wang, Tao Guo, Lijie Zhu, and Sheng Wu. All authors contributed to the article and approved the submitted version.

## CONFLICT OF INTEREST STATEMENT

There has no conflict of interest to be declared.

## AVAILABILITY OF DATA AND MATERIALS

The public data used in the current study were obtained from UCSC Xena (https://xena.ucsc.edu/), GEO (https://www.ncbi.nlm.nih.gov/geo/), Mendeley Data (https://data.mendeley.com/), and TISCH database (http://tisch.comp‐genomics.org). High throughput sequencing data were available from the corresponding authors upon request.

## ETHICS APPROVAL AND CONSENT TO PARTICIPATE

The ethics committee of the Affiliated Hospital of Jiangnan University reviewed and approved the studies involving human participants and experimental animal studies. Each patient signed an informed consent form. All experiments were performed in accordance with the Declaration of Helsinki and all methods were reported in compliance with ARRIVE guidelines.

## CONSENT FOR PUBLICATION

Not applicable.

## Supporting information

Supplementary Material

## Data Availability

The public data used in the current study were obtained from UCSC Xena (https://xena.ucsc.edu/), GEO (https://www.ncbi.nlm.nih.gov/geo/), Mendeley Data (https://data.mendeley.com/), and TISCH database (http://tisch.comp‐genomics.org). High throughput sequencing data were available from the corresponding authors upon request.
