# Supplementary material for: A tumour‐associated macrophage‐based signature for deciphering prognosis and immunotherapy response in prostate cancer
Source: IET Syst Biol. 2024 Aug 13;18(5):155–71. doi: 10.1049/syb2.12097 (PMC11490193; doi:10.1049/syb2.12097)
Supplement: Supplementary file 1 — Supplementary Material [file SYB2-18-155-s001.docx]

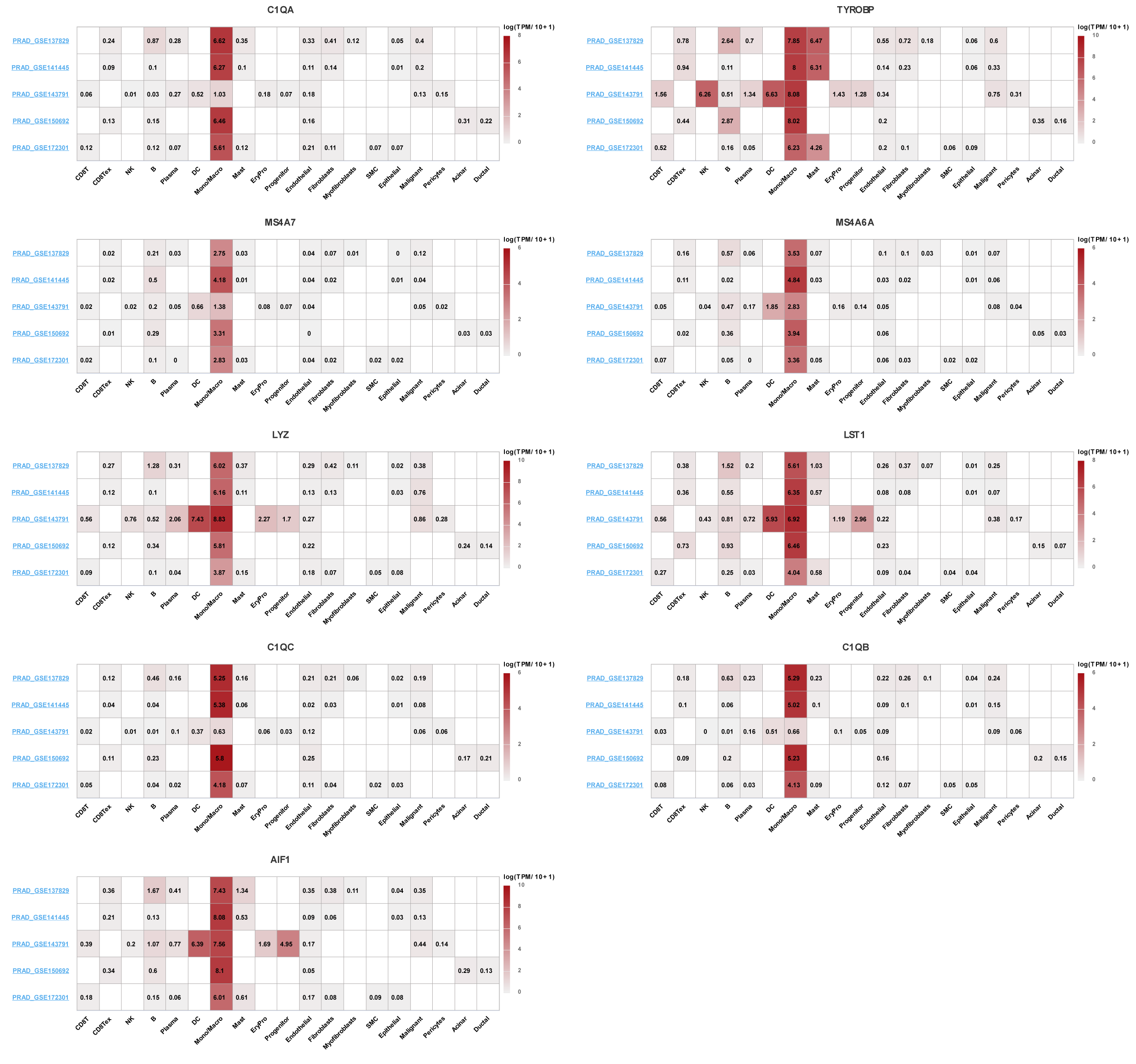


**Supplementary Figure 1** | Distribution of macrophage-specific genes in the TISCH database.


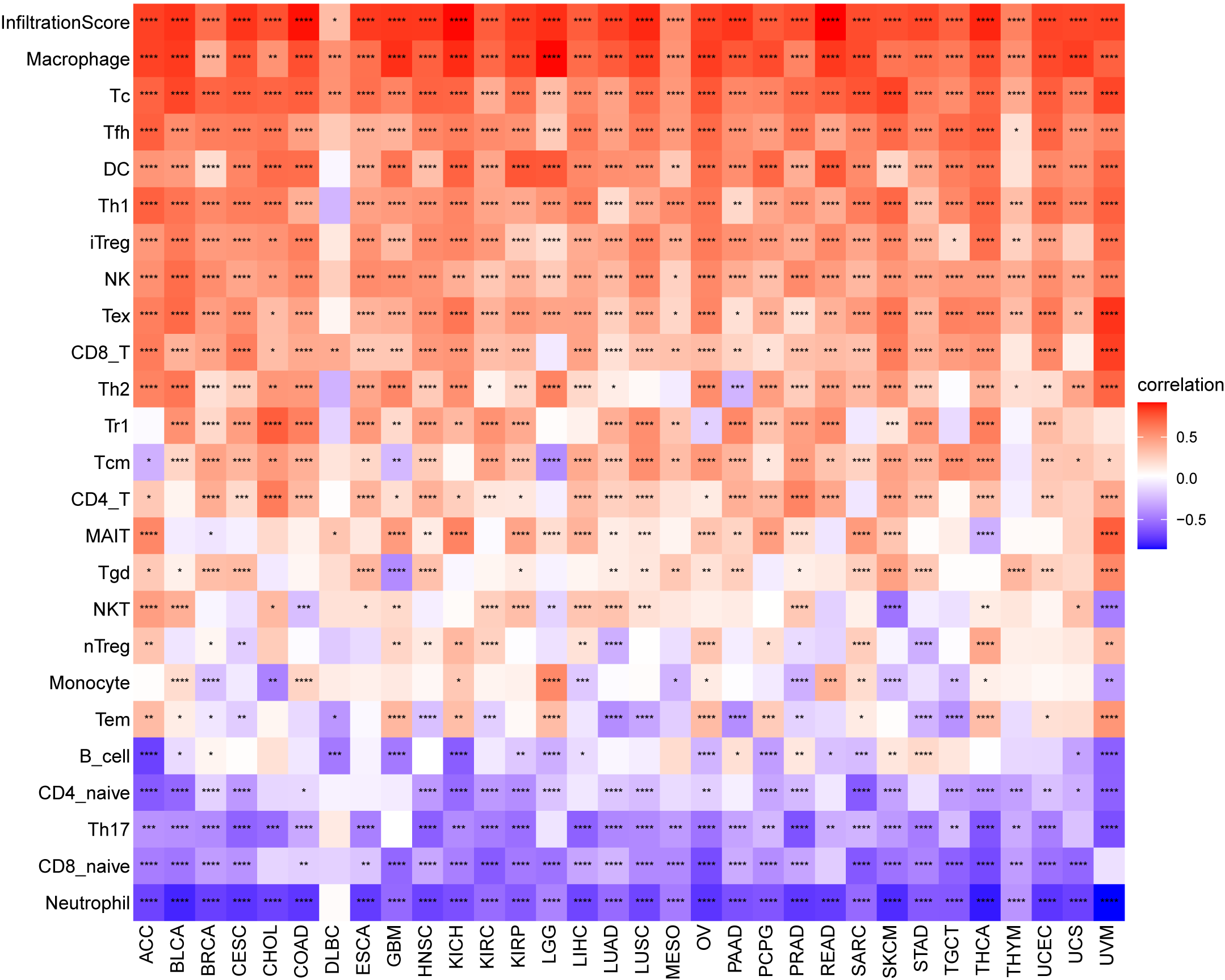


**Supplementary Figure 2** | Correlation analysis between scores composed of macrophage-specific genes and immune cell infiltrations.


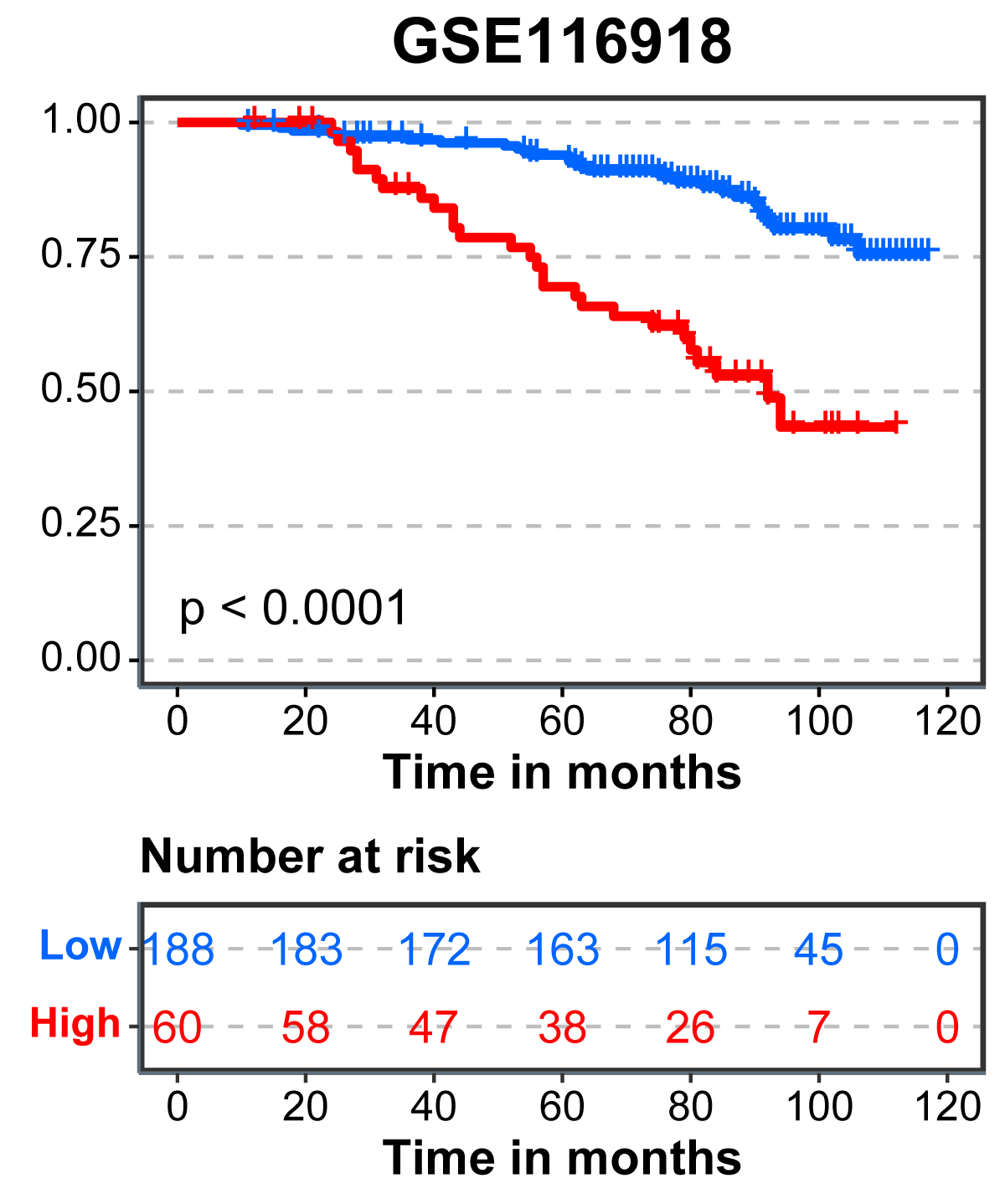


**Supplementary Figure 3** | Kaplan–Meier analysis showing prognostic differences between the high-MRS and low-MRS groups in GSE116918 cohort.


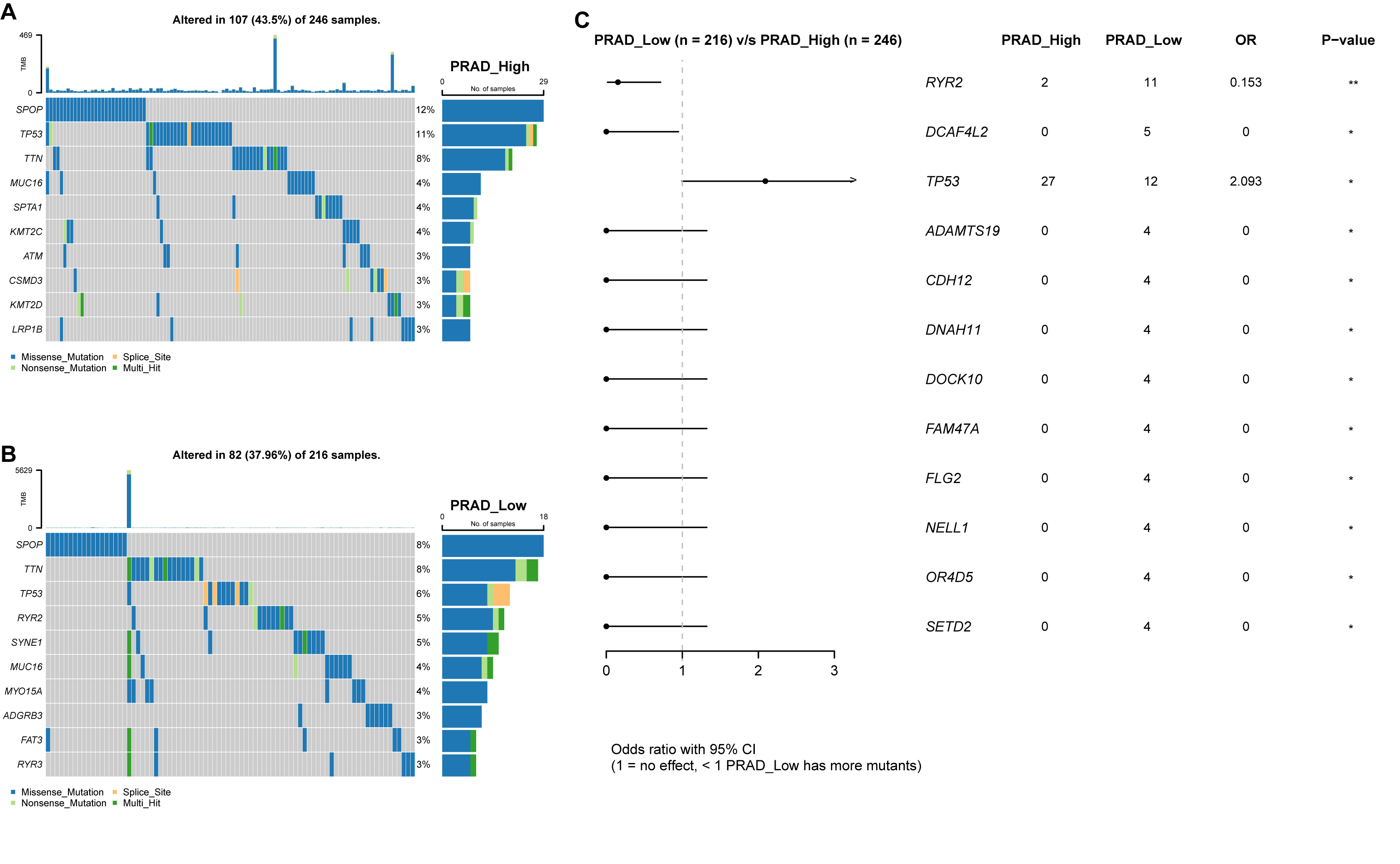


**Supplementary Figure 4** | Mutation landscape in different MRS groups. **(A-B)** Waterfall Plot displaying the top mutated genes between high-MRS and low-MRS groups. **(C)** The most significantly different mutant genes.


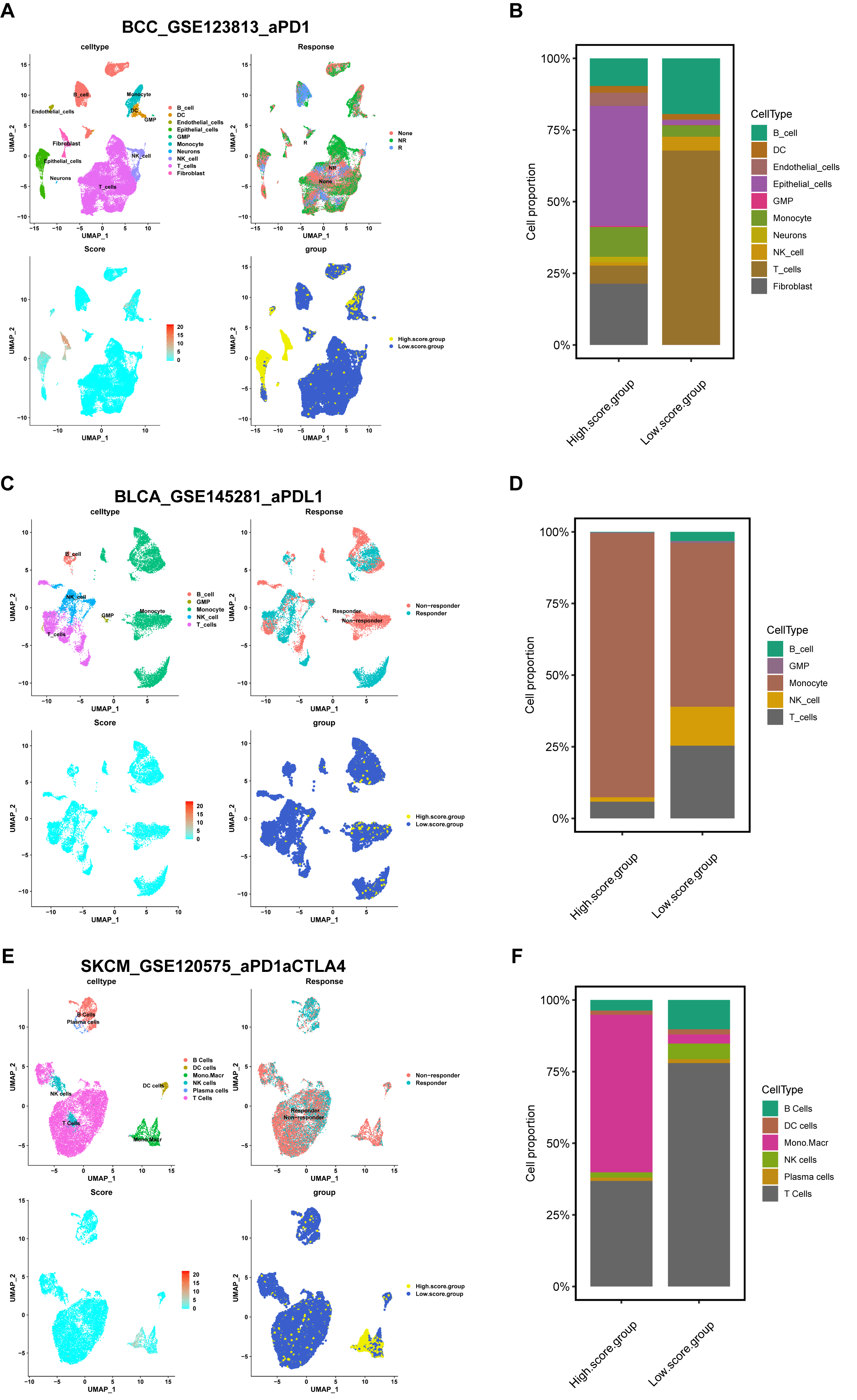


**Supplementary Figure 5** | Effective predictive power of MRS for immunotherapy response in single-cell data. **(A-F)** Distribution of MRS in each cell and different cellular components between high-MRS and low-MRS groups (GSE123813, GSE145281, and GSE120575).


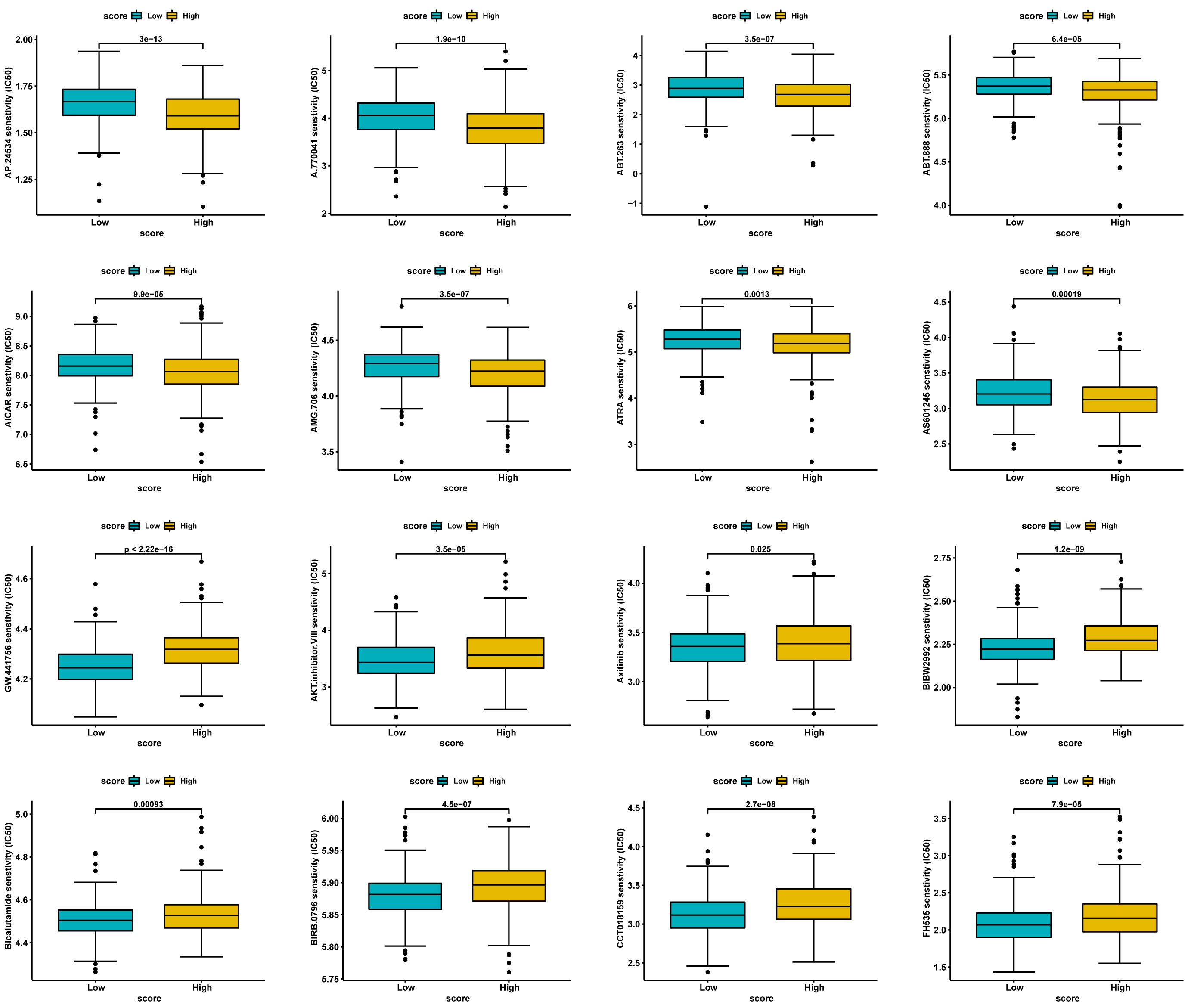


**Supplementary Figure 6** | Differences in drug sensitivity between low-MRS and high-MRS groups.
